# Timing of drainage tube removal and flap recovery after osmidrosis surgery: a case series

**DOI:** 10.1093/jscr/rjag083

**Published:** 2026-02-19

**Authors:** Olivia Cheng, Wen-Tsao Ho

**Affiliations:** International Bilingual School at Hsinchu Science Park, No. 300, Jieshou Rd., East District, Hsinchu City 30078, Taiwan; Department of Dermatology, Ho Wen Tsao Skin Clinic, No. 179, Section 2, Wenhua 3rd Rd., Linkou District, New Taipei City 244, Taiwan

**Keywords:** osmidrosis, axillary hyperhidrosis, Penrose drain, flap perfusion, postoperative management, case series

## Abstract

The optimal timing for drainage tube removal after osmidrosis surgery is uncertain. This case series investigates the relationship between drainage duration and flap recovery following osmidrosis surgery. Thirty patients (60 axillae) underwent minimally invasive liposuction-assisted curettage combined with the blunt-scissor technique using two 5-mm incisions at the anterior and posterior axillary borders, with one 5-mm Penrose drain inserted per incision. Patients were divided into three groups according to the postoperative day (POD) of drain removal—POD 3, 6, and 9. Tie-over dressings were removed uniformly on POD 3, and white dermal erosions were recorded as indicators of transient ischemia. White erosions occurred in 3, 1, and 0 axillae, respectively, suggesting that maintaining drainage for at least 6 days optimizes flap perfusion and minimizes ischemic dermal changes after osmidrosis surgery.

## Introduction

Osmidrosis, a socially distressing dermatologic condition caused by overactive apocrine glands, is treated through various methods including surgical excision, trimming, liposuction-curettage, and laser ablation [1–3]. Surgical methods remain the most effective, achieving the lowest recurrence rates. Yet, they present challenges such as hematoma, necrosis, and prolonged healing [4–6].

Refinements in minimally invasive surgery, such as tumescent liposuction with dermal curettage [2], small-incision trimming [7], and power-assisted curettage [8], have improved cosmetic results and reduced complications. However, consensus among surgeons regarding postoperative management, specifically the duration of drainage, has not been established. Early removal can result in subflap fluid accumulation and compromised microcirculation, whereas prolonged drainage may increase infection risk [9]. This study evaluates how drainage duration influences flap perfusion and recovery outcomes using white dermal erosions as indicators of transient ischemia.

## Case presentation

### Patient selection

Between January and June of 2025, 30 consecutive patients (60 axillae) with primary osmidrosis underwent subdermal rotary curettage under local anesthesia. The participants consisted of 18 female and 12 male patients between 18 and 36 years of age. All participants provided written informed consent for surgery.

Inclusion criteria: Clinical diagnosis of primary osmidrosis with moderate to severe symptoms; No previous axillary surgery.

Exclusion criteria: Smoking or chronic alcohol use; Diabetes mellitus or autoimmune disease; Active infection, dermatitis, or previous axillary scarring.

### Surgical technique and drainage placement

**Figure 1:**
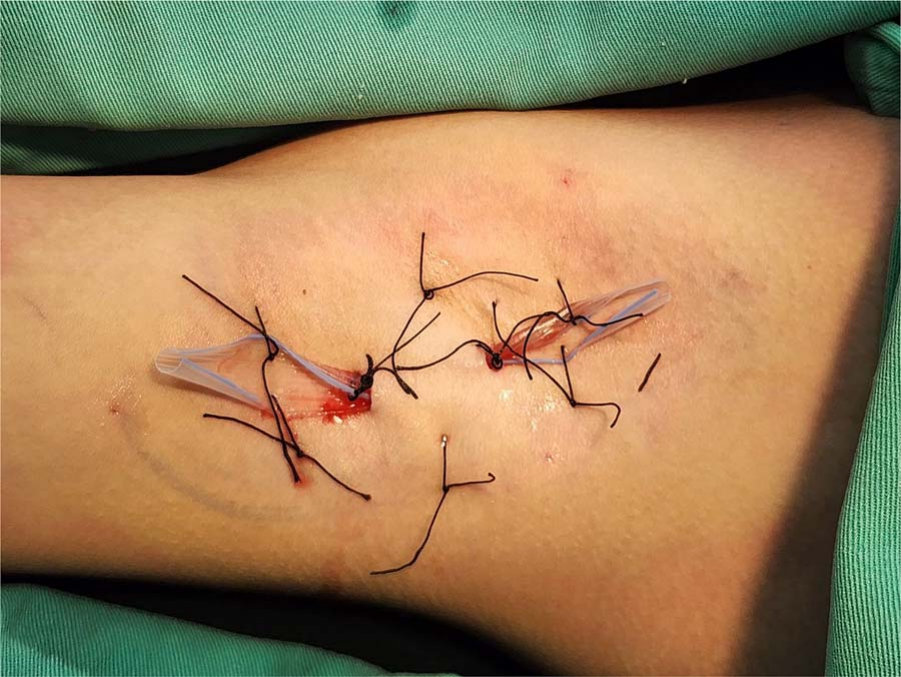
Bilateral axillary drains (5-mm Penrose) secured with 3–0 silk sutures via anterior and posterior incisions, with quilting sutures (3–0 silk) obliterating dead space.

All procedures used the minimally invasive liposuction-assisted curettage combined with the blunt-scissor method [1]. Two 5-mm incisions were made at the anterior and posterior axillary borders. After infiltration with tumescent solution (200 ml normal saline, 20 ml 2% lidocaine, and 1 ml 1:1000 epinephrine), the subdermal flap was elevated and the apocrine glands were removed via three controlled maneuvers. Two 5-mm Penrose drains were placed in each axilla—one anterior, one posterior—and secured with 3–0 silk sutures. Quilting sutures (3–0 silk) and tie-over dressing were applied to obliterate dead space and promote flap adherence (Fig. 1). Quilting sutures and tie-over dressing were removed on postoperative day (POD) 3. After removal, patients were instructed to perform daily wound care by changing sterile gauze and applying topical antibiotic ointment. Each patient submitted daily photographs of the drainage area and gauze exudate using a secure mobile application, allowing clinicians to monitor healing progression and determine the appropriate timing for drain removal. Drainage removal was performed on POD 3, 6, or 9 according to group arrangement (Table 1).

**Table 1:** Incidence of white dermal erosions by Penrose drain removal timing (POD 3, 6, or 9; n = 10 per group)

Group	Drain removal day	Cases (n)	White dermal erosions (n)	Typical erosion size
A	POD 3	10	3	Left 1 × 1 cm, Left 1.5 × 1 cm + 0.5 × 0.5 cm, Right 0.5 × 1 cm
B	POD 6	10	1	Right 0.5 × 0.3 cm
C	POD 9	10	0	—

Erosion size indicates the largest lesion measured. Grouping by drainage duration.

**Figure 2:**
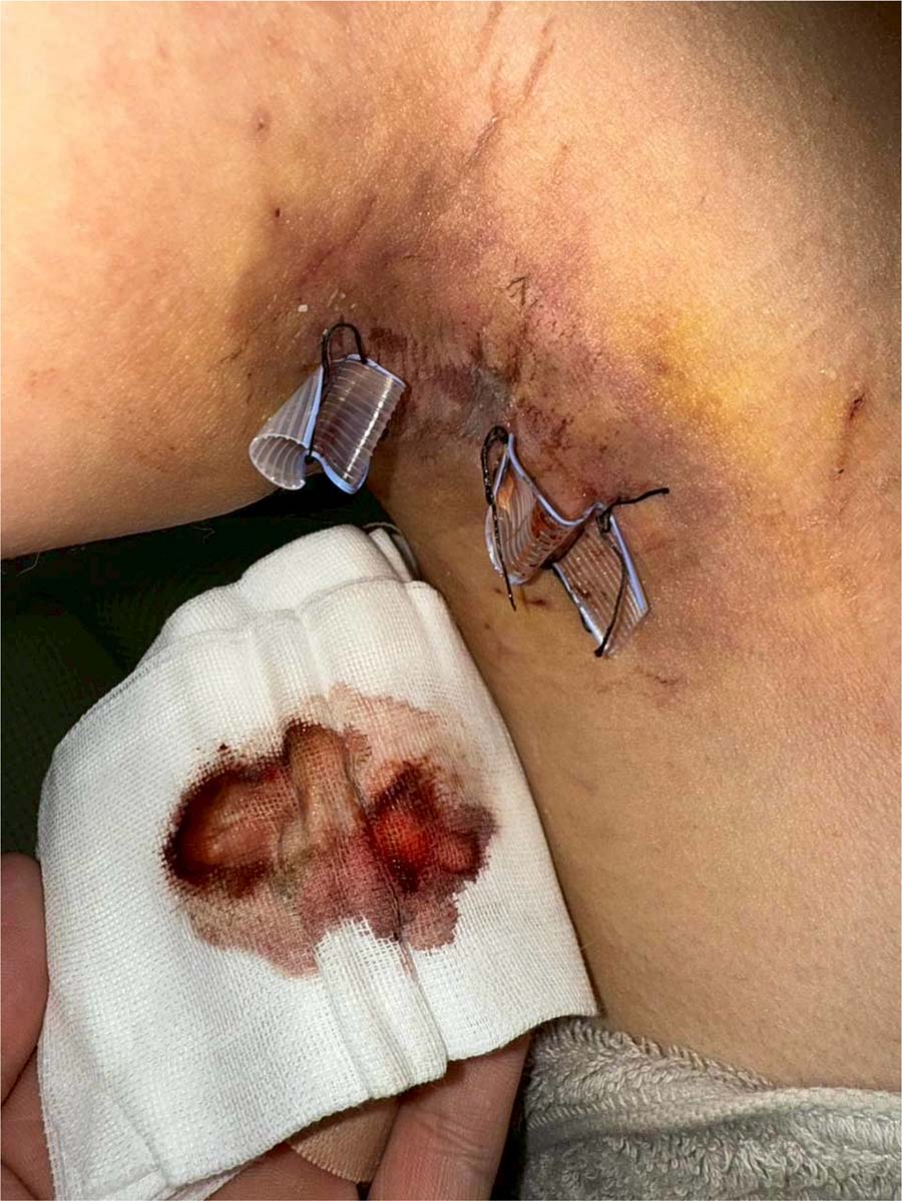
Daily postoperative photograph showing Penrose drains *in situ* with serosanguinous output on gauze, used for monitoring drainage amount and flap condition.

**Figure 3:**
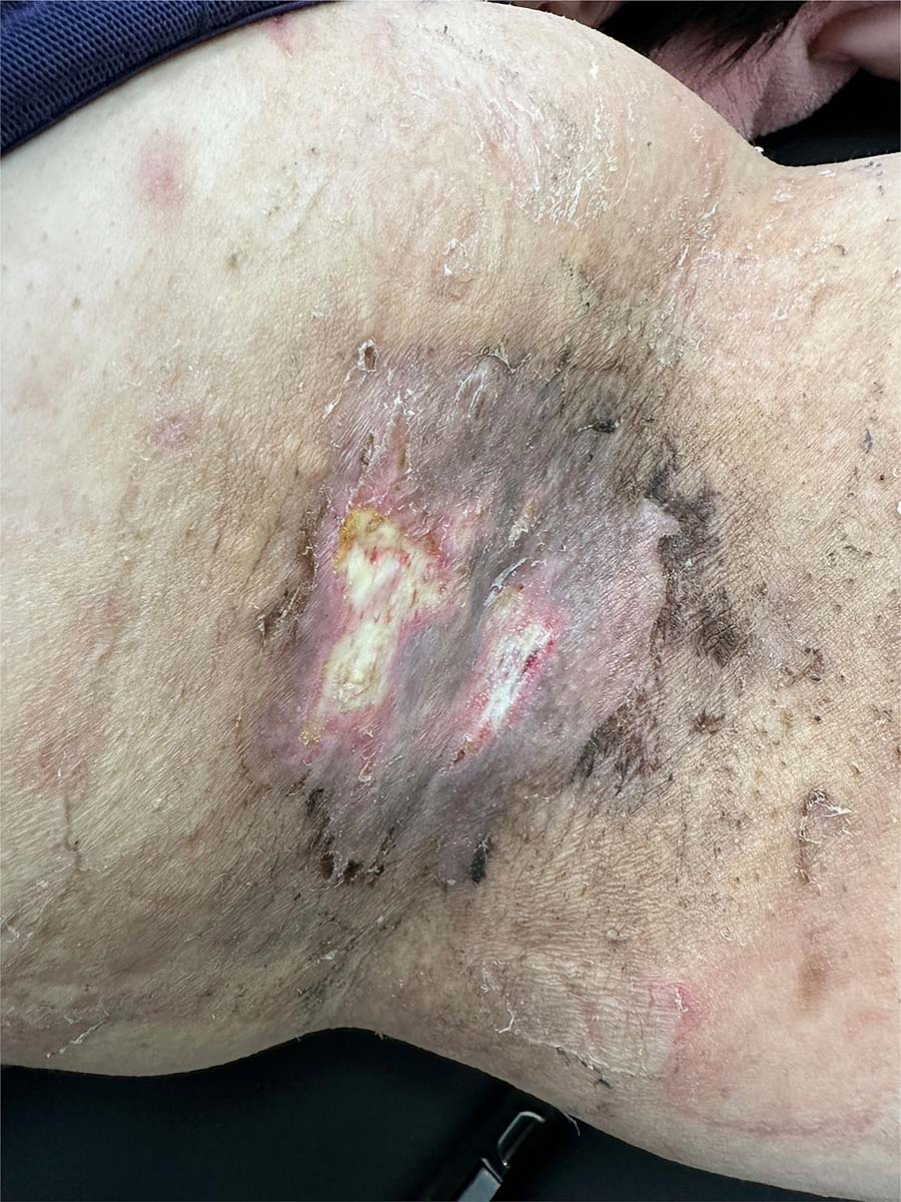
One case showing 1.5 × 1.0 cm and 0.5 × 0.5 cm superficial white dermal erosions on the left axilla 2 weeks after early drain removal (POD 3).

Postoperative wound and drainage status were monitored daily through patient-submitted photographs to assess drain output, flap color, and local swelling (Fig. 2). Superficial white dermal erosion occurred most frequently in the POD 3 group (three cases), less frequently in POD 6 (one case), and was not observed in POD 9. Representative case from Group A (POD 3 removal) showing two areas of white dermal erosions (1.5 × 1.0 and 0.5 × 0.5 cm) on the left axilla. The lesions exhibited partial epithelial loss without necrosis, indicating transient ischemic change after early drain removal (Fig. 3). Superficial white dermal erosions were defined as localized epidermal whitening without necrosis. All patients were followed for 3 weeks, and no infection, hematoma, or flap necrosis occurred.

## Discussion

To date, no study has specifically investigated the optimal timing for drain removal following minimally invasive osmidrosis surgery. This case series addresses this evidence gap by evaluating how drainage duration influences flap perfusion and recovery outcomes. The results demonstrate that drainage duration directly determines flap survival: early removal on POD 3 consistently resulted in subflap exudate accumulation, elevated intradermal pressure, and transient ischemic whitening, whereas maintaining drainage until POD 6–9 allowed progressive microcirculatory recovery and complete resolution of dermal pallor.

The significance of drainage is further underscored by the absence of drains in several reported series, where higher rates of flap necrosis and haematoma liquefaction were observed despite similar surgical techniques [6, 10]. These findings suggest that the lack of continuous decompression, rather than the extent of glandular removal alone, may be a primary contributor to ischemic complications. The dual-drain system employed in this study achieved mechanical stabilization comparable to quilting sutures [4], but with the added benefit of sustained negative pressure, effectively preventing seroma-induced microvascular compromise.

Adequate drainage and controlled compression remain key principles in flap management [9]. Premature drain removal elevates subflap pressure, triggering a cascade of venous congestion, hematoma liquefaction, and delayed necrosis—mechanisms directly observed in our POD 3 cases [11]. Conversely, low-pressure drainage sustained for at least 6 days balances exudate evacuation with infection control, while preserving endothelial integrity and tissue perfusion [4, 5].

Although advanced techniques such as tumescent liposuction with curettage [2, 3, 7, 8], pinch-and-turn-over approaches [12], and power-assisted instrumentation [8] have reduced incision length and recurrence, their efficacy is contingent upon concomitant prolonged drainage. Systematic reviews confirm that surgical excision yields the lowest recurrence rates [6], yet complication profiles are significantly improved when drainage is prioritized over early mobilization. Prolonged antiperspirant use may further mitigate postoperative erythema by reducing baseline inflammation [13].

This study establishes POD 6–9 as the evidence-based window for safe drain removal and highlights routine drainage as a non-negotiable component of modern osmidrosis surgery. Future randomized trials should focus on drainage duration as the primary variable, rather than continuing to compare surgical tools in the absence of standardized decompression protocols.

## Conclusion

Early drain removal (< 6 days) after osmidrosis surgery may compromise flap microcirculation, resulting in transient ischemia and delayed epithelial recovery.

Maintaining bilateral 5-mm Penrose drains for at least 6 days provides continuous exudate evacuation, minimizes interstitial pressure, and optimizes flap perfusion and healing outcomes.

Although this study adopted a fixed design to allow clear comparison between postoperative days, clinical application can be individualized.

In daily practice, patients may record gauze exudate levels via smartphone applications and transmit the images to clinicians for real-time assessment, allowing flexible and safer decisions regarding drain removal timing.
